# An On-Line Cross-Sectional Questionnaire to Assess Knowledge of COVID-19 Pandemic among Citizens Tested for the SARS-CoV-2 Virus in Quito and Ibarra, Ecuador

**DOI:** 10.3390/ijerph18041691

**Published:** 2021-02-10

**Authors:** David Ortega-Paredes, Jeannete Zurita, Camilo Zurita, Paula Leoro-Garzón, Galo Leoro-Monroy, César Marcelo Larrea-Álvarez, Karen Loaiza, Esteban Fernandez-Moreira, Gabriel Molina-Cuasapaz, Marco Larrea-Álvarez

**Affiliations:** 1 Unidad de Investigación en Enfermedades Transmitidas por Alimentos y Resistencia a los Antimicrobianos (UNIETAR), Facultad de Veterinaria, Universidad Central del Ecuador, Quito 170129, Ecuador; daortegap@uce.edu.ec; 2 Education Unit, Life Science Initiative, Quito 170607, Ecuador; cmla88@hotmail.com (C.M.L.-Á.); karenloaizaconza@gmail.com (K.L.); 3 Zurita & Zurita Laboratorios, Biomedical Research Unit, Quito 170104, Ecuador; jzurita@zuritalaboratorios.com (J.Z.); camiloszuritas@zuritalaboratorios.com (C.Z.); 4 Departamento de Biología Molecular, Laboratorio Clínico e Inmunológico Inmunolab, Quito 170403, Ecuador; paula.leorog@gmail.com (P.L.-G.); gleoro@hotmail.com (G.L.-M.); 5 Facultad de Ciencias de la Salud, Escuela de Medicina, Universidad de las Américas, Quito 170517, Ecuador; 6 Facultad de Ciencias Agropecuarias y Recursos Naturales, Carrera de Medicina Veterinaria, Universidad Técnica de Cotopaxi, Latacunga-Cotopaxi 050101, Ecuador; edie.molina7278@utc.edu.ec; 7 School of Biological Science and Engineering, Yachay-Tech University, Hacienda San José, Urcuquí-Imbabura 100650, Ecuador

**Keywords:** COVID-19, knowledge, Ecuador, survey, SARS-CoV-2, private laboratory

## Abstract

Several public health measures have been implemented to contain the SARS-CoV-2 outbreak. The adherence to control measures is known to be influenced by people’s knowledge, attitudes and practices with regard to the disease. This study aimed at assessing COVID-19 knowledge in individuals who were tested for the virus. An online cross-sectional survey of 32 items, adapted to the national context, was conducted among 1656 Ecuadorians. The mean knowledge score was 22.5 ± 3 out of 28, with significant differences being observed with regard to educational attainment. People with postgraduate training scored higher than those with college, secondary and elementary instruction. Indeed, multiple linear regression revealed that lower scores were associated significantly with the latter three levels of education. Interviewees were knowledgeable about the symptoms, detection, transmission and prevention of the disease. However, they were less assertive regarding the characteristics of the virus as well as the usefulness of traditional and unproven treatments. These outcomes indicated a lack of knowledge in fundamental aspects of virus biology, which may limit the effectiveness of further prevention campaigns. Conclusively, educational and communicational programs must place emphasis on explaining the basic molecular characteristics of SARS-CoV-2; such information will certainly contribute to improve the public’s adherence to control measures.

## 1. Introduction

The new coronavirus disease, COVID-19, has been declared a pandemic and could be considered one of the most important global health calamities of recent years [1]. The causative agent of COVID-19 is the new coronavirus strain called SARS-CoV-2 (severe acute respiratory syndrome coronavirus 2) [2]; coronaviruses are positive-sense single-stranded RNA viruses that are divided, based on their antigenicity, into four groups: alpha, beta, gamma and delta [3]. All coronaviruses groups infect mammals, but alpha and beta coronaviruses can indeed infect humans, eliciting a variety of conditions ranging from the common cold to bronchiolitis and pneumonia [4]. Infection by SARS-CoV-2 causes predominantly upper respiratory tract symptoms, but lower respiratory tract infections have been also been detected [5,6]. Currently, it is still not yet determined if people who have recovered from the disease are protected from a second infection [7]. Additionally, there is diverse evidence about the severity and dissemination of the virus led by its genetic variants, as well as their implications in the vaccine development [8]. As of 25 May 2020, the estimated SARS-CoV-2 case fatality rate is around 6.4% [9], mainly affecting the elderly and people with comorbidities (pulmonary diseases, diabetes), although to a lesser extent than SARS-CoV (around 9.5%) and MERS-CoV (around 35%) [10]. SARS-CoV-2 differs from its predecessors as it is highly contagious; this virus is easily transmitted among humans via direct contact or respiratory droplets, and this has led to its rapid dissemination and the enormous number of registered infections [11,12]. Consequently, SARS-CoV-2 has proliferated faster than public health measures could contain, especially in countries where medical facilities are not sufficiently equipped; they reach their maximum capacity almost immediately, leading to the collapse of health care systems and the rise of new COVID-19 cases and related deaths [13]. Despite the fact that such devastating effects have been observed worldwide, this disease is more likely to affect countries with limited systems, such as those belonging to the Global South, especially Latin America and Sub-Saharan Africa [14,15].

Ecuador has been severely affected by the pandemic, with confirmed cases in all 24 provinces [16]. The case fatality rate reported for the country is around 6.6% with 13,994 deaths out of 209,758 cases until December 28 of 2020 [17]. However, this value might be underestimated, as 2020 has yielded more than 30,000 deaths, compared to what was reported during the same period in 2019 [18]. 

People’s adherence to prevention and control campaigns is known to be influenced by their knowledge, attitudes and practices as regards COVID-19 [19,20]. Common misunderstandings, with regards to the treatments, transmission, prevention, characteristics and origin of the virus, circulate frequently on social media, and such misinformation could undoubtedly contribute to the overall impact of the pandemic on the public [21]. In Ecuador, for instance, important misunderstandings have been disseminated in such a way, including misconceptions regarding the development of vaccines, viral mutations, the origin of the virus or the use of sea water as a potential treatment [22,23]. Multiple reports have focused on assessing the knowledge, attitudes and practices among individuals of different communities [24,25,26,27]. These investigations have all made use of a similar survey that focuses on determining knowledge about symptoms, transmission and prevention. These reports have thus recommended the expansion of the questionnaire in order to get a better understanding of people’s knowledge about the biology of the virus, along with their assessment of traditional and unproven treatments. In this investigation, we adapted the aforementioned form to the national context and included supplementary queries dealing with the virus characteristics, its detection and treatments. Studies assessing the knowledge of contagious diseases provide reference information that could be useful to define the type of measures required to change misconceptions about the related pathogens. The data gathered by these investigations would be helpful for providing better insights, and so address deficient knowledge about diseases. In addition, this information could be applied for developing preventive strategies and promoting health programs.

The developed questionnaire was used to assess knowledge of COVID-19 in individuals tested for SARS-CoV-2 in the cities of Quito and Ibarra; this subpopulation could be considered of importance as they might be actively involved in the dissemination of the virus. Furthermore, we sought to explore an approach in which interviewees were provided with the correct answers after responding to each question. In this way, diagnostic laboratories could contribute to increasing knowledge about this infectious disease, and thus correct common misconceptions recognized among the public. The present survey provides valuable information pertaining to COVID-19 literacy in Ecuadorians at potential risk of contracting and spreading the disease, and such knowledge must be considered when developing preventive strategies and control programs aimed at reducing the impact of the current pandemic.

## 2. Materials and Methods

This investigation was conducted in Quito, Ecuador from the 25 August to 8 September 2020 in two laboratories: Zurita&Zurita Laboratorios and Laboratorio Clínico Inmunolab. These laboratories are currently authorized by the Ecuadorian Department of Public Health for carrying out SARS-CoV-2 PCR tests. The sampled population included individuals with suspected symptoms of COVID-19, and people with no apparent symptoms but who were required to be tested for job-related reasons. Verbal consent was provided by all surveyed individuals after being informed about the scope of the study; the questionnaire was voluntary and anonymous, and due to the low-risk nature of the investigation, approval from an ethical committee was not required. Individuals were selected based on non-random criteria, since the present research was aimed at developing an initial understanding of COVID-19 knowledge in this small population. As this investigation did not intend to test any hypothesis about a broader population, the survey was conducted using voluntary response samples. Additionally, we referred to the checklist for reporting results of internet e-surveys (CHERRIES) as a way of increasing the reliability of the form (Table S1). The link to the online questionnaire was delivered, in Spanish, via electronic mail; addresses were provided voluntarily by the participants after being sampled. The interviewees were instructed to complete the questionnaire by following the instructions, with correct responses being revealed after answering each question. 

The survey (Table S2) consisted of 6 sections with a total of 32 questions. The first four involved the demographic data, which included age, gender, educational attainment (postgraduate, college, secondary and elementary) and city of residence. From this point, questions were numbered from 1 to 28. The second part consisted of questions about symptoms. These included differences between flu and COVID-19 symptoms. Another question dealt with the impact on high-risk individuals in relation to symptoms and their comorbidities. The third section covered knowledge with regards to PCR tests versus rapid tests; both precision and usefulness in diagnosis were considered. The fourth section was about treatments, with a special emphasis on the use of alternative, natural and home remedies. The fifth part consisted of questions about transmission, while the sixth section included questions about prevention strategies aimed at avoiding the transmission of the virus. The final part was envisioned to evaluate knowledge about the SARS-CoV-2 virus, and was composed of six queries. Additionally, at the end of the questionnaire, people were offered a segment wherein they could share their thoughts, impressions and recommendations.

Questions 1–3, 6, 9–11, 13, 15–18 and 20 were based on a previous investigation that assessed knowledge, attitudes and practices (KAP) among Malaysians [26]; this study used a previously developed survey aimed at measuring KAPs in Chinese residents [24]. Nevertheless, additional questions were formulated based on common misconceptions in the area, as we wanted to gain a deeper understanding of the participants’ knowledge about the virus, and its detection, treatments and prevention in the local context. From these, Q4 and Q5 were included as a gauge to assess knowledge of detection techniques, whereas Q7 and Q8 did so as a way of estimating the awareness of traditional and unproven treatments. Q12 and Q14 aimed at evaluating potential means of transmission not included in the original investigations, while Q19, Q21 and Q22 included prevention approaches not addressed in the mentioned studies. Finally, questions Q23–28 dealt with the virus’ origins, characteristics, and induced-mortality in the country. Sections regarding symptoms (Q1–3), detection (Q4 and 5), treatment (Q6–8), transmission (Q9–14) and prevention (Q15–22) included only “True or False” (T/F) questions (“true”, “false” and “not sure” were provided as options), while the ones concerning knowledge of the virus were divided between T/F (Q23 and 24) and multiple-choice questions (Q25–28), with the latter postulating a unique correct response. Accurate answers were assigned one point, whereas incorrect/not sure responses were not. Scores ranged from 0 to 28, with better results indicating stronger knowledge.

The data analyses were carried out using MATLAB^®^ version 9.9.9341360 (MathWorks, Natick, MA, USA) (R2016a); figures were obtained using Python’s plotting library, Matplotlib 3.0.3 (Python Software Foundation, Fredericksburg, VA, USA). Descriptive analyses relied on frequencies and percentages, while differences within demographic groups were assessed using independent *t*-test and independent one-way analysis of variance (ANOVA), along with a Tukey post-hoc. Chi-square was employed for determining potential relationships between demographic variables and common misconceptions regarding treatments and the characteristics of the virus. Multiple linear regression analysis was utilized to determine potential factors related to knowledge, using demographic categories as independent variables and scores as the dependent variable. Statistical significance was set at *p* < 0.05. The Cronbach alpha coefficient was calculated in an attempt to assess the consistency of the novel variables (15 queries). The value obtained for knowledge was 0.65. It has been proposed that coefficients ranging from 0.6 to 0.7 are considered adequate and reliable [26,28]. As such, the questions utilized herein appear acceptable for the purpose of measuring knowledge of COVID-19.

## 3. Results

A total of 1656 patients answered the questionnaire voluntarily (Table S3). From these, 1596 surveys were filled correctly and thus used for the analyses. The demographic data of participants are shown in Table 1.

More than half of the population (868, 54.4%) were stated to be between 30 and 49 years of age, while the rest were divided among people between 18 and 29 years of age (362, 22.7%), older than 50 (356, 22.3%) and younger than 18 years (10, 0.6%), with women (854, 53.5%) being more common than men (742, 46.5%). Out of the total, 851 individuals (53.3%) declared to have attended university, whereas 396 (24.8%) mentioned having completed a post-graduate program. In total, 321 patients (20.1%) asserted completing high school, and only 28 (1.8%) stated they had completed primary education. The majority (1261, 79%) claimed to reside in Quito and Ibarra; cities with less than thirty counts were classified as “other cities’’. People residing in different areas did so in Santo Domingo de los Tsáchilas (56), Guayaquil (7 counts) and Portoviejo (3). Overall, the mean score was 22.5 ± 3 with significant differences only being observed for educational attainment (*p* < 0.001) (Table 1). The post-hoc analysis revealed that the score obtained by people with postgraduate education was significantly higher than that of people belonging to the other categories (*p* < 0.001). Similarly, college-instructed individuals scored higher than those with secondary and elementary education (*p* < 0.01). No differences were observed between the two latter categories (Table 1). Multiple linear regression (R^2^ = 0.0911) revealed that only college instruction or lower (vs. postgraduate education, β: −1.205–3.427, *p* < 0.001) was associated with lower knowledge scores (Table 2). 

**Figure 1:**
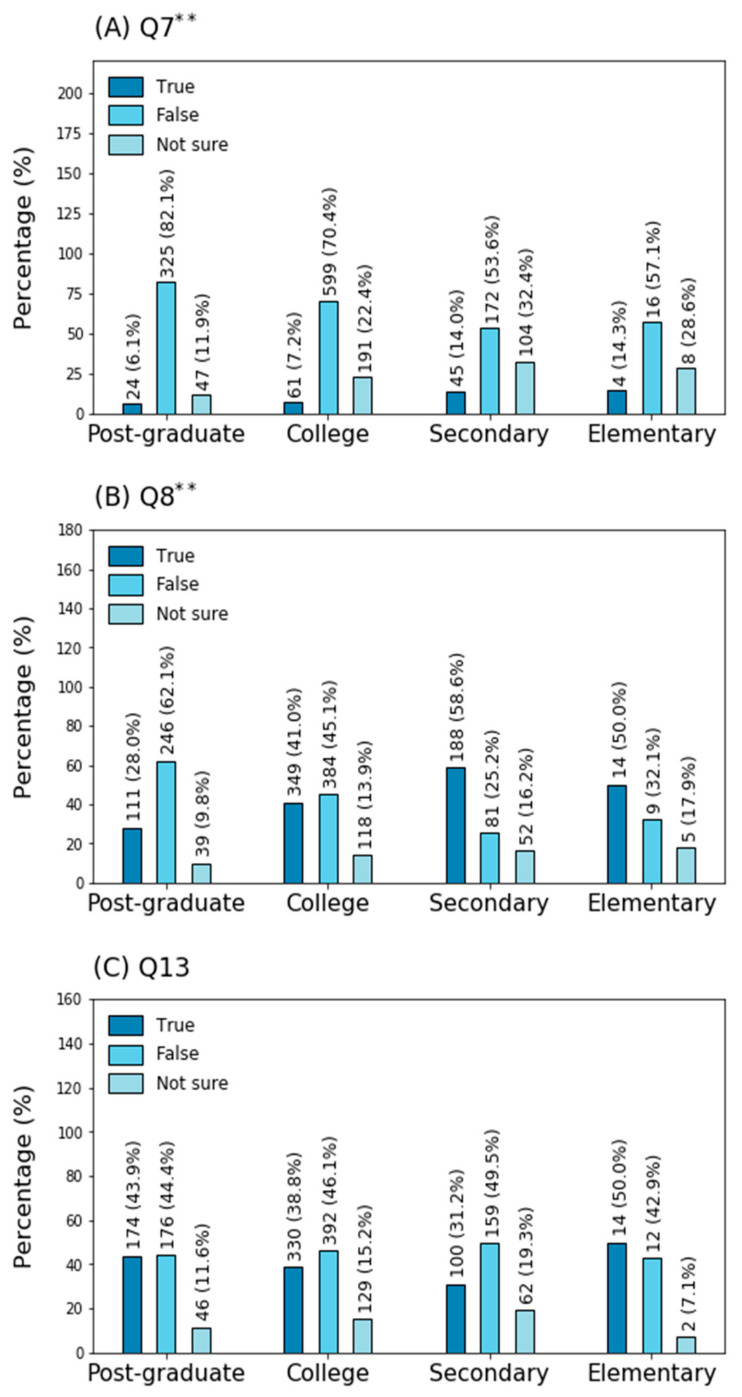
Comparison of response percentages among educational groups: (**A**) Question 7; (**B**) Question 8; (**C**) Question 13. ** Chi-square values significant at *p* < 0.01.

In general, people were familiar with the main clinical symptoms and did recognize that severe cases develop under particular conditions. However, participants were not so confident regarding symptoms less commonly associated with COVID-19 than with the common cold, as only 58% answered the related question correctly (Table 3). Overall, surveyed individuals were aware of the specificity differences between the tests utilized for virus detection, as more than 90% agreed that PCR is the most accurate technique (Table 3). On the other hand, participants were less assertive about treatments, with 21% of them not being sure about chloroquine dioxide safety, and 8.4% accepting that it is a secure treatment (Table 3). In fact, people with postgraduate backgrounds were more likely to consider this drug unsafe (80%), and such assessment proved to be less popular among people with college (70.4%), secondary (53.6%) and elementary educations (57.1%) (Figure 1A).

Furthermore, only 45.1% of the population agreed that traditional medicine cannot be used for fighting the virus and treating the disease (Table 3). Again, postgraduate-educated individuals seemed more reluctant to agree with the common belief that honey, garlic, ginger or eucalyptus can be used to fight the disease, as 62.1% did not agree with such statement. College-instructed individuals were more dubitative, as only 45.1% claimed that these options could be used as treatments, while participants with secondary (58%) and elementary (50%) education were more inclined to trust such alternatives (Figure 1B). Participants stating the efficacy of these treatments were associated with a lower educational status; chi-squared analysis revealed significant differences only by educational attainment. Despite the obvious confusion, 95.2% of the population acknowledged that no effective cure is available yet (Table 3). The interviewees, in general, were aware that the virus could be transmitted to others, even if the patients did not present fever, and that transmission occurs via respiratory droplets of infected individuals. Moreover, the participants knew that people can be infected despite not showing any clear symptoms of illness, as more than 93% responded correctly to the associated queries. Nevertheless, they were less confident regarding the role of both wild and domestic animals in infection, with less than 80% answering accurately. In addition, less than 50% of participants recognized that the virus is airborne (Table 3); such a pattern was observed independently of the educational level (Figure 1C). In general, over 90% of participants agreed with preventive measurements such as avoiding crowded places and public transportation, the isolation and treatment of infected people, as well as the correct use of gloves, masks and protective screens as effective ways to reduce the spreading of the virus. Nonetheless, only 84% asserted that returning to the workplace without being tested is appropriate (Table 3). 

**Figure 2:**
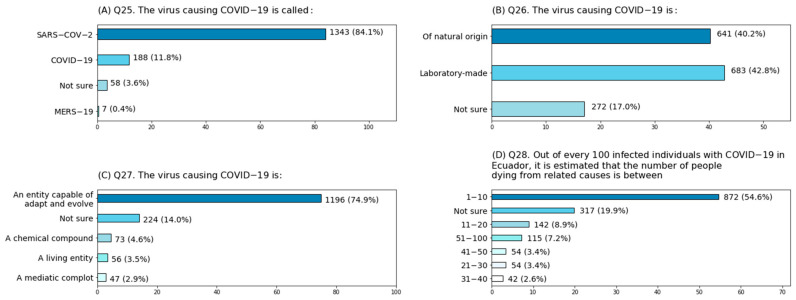
Response percentages for multiple-option questions. (**A**) Question 25; (**B**) Question 26; (**C**) Question 27; (**D**) Question 28.

Knowledge about the virus was less common than that assessed in the other categories. The only questions in which more than 70% of the population answered correctly dealt with the name of the virus (84%) and its characteristics (75%) (Figure 2). 

**Figure 3:**
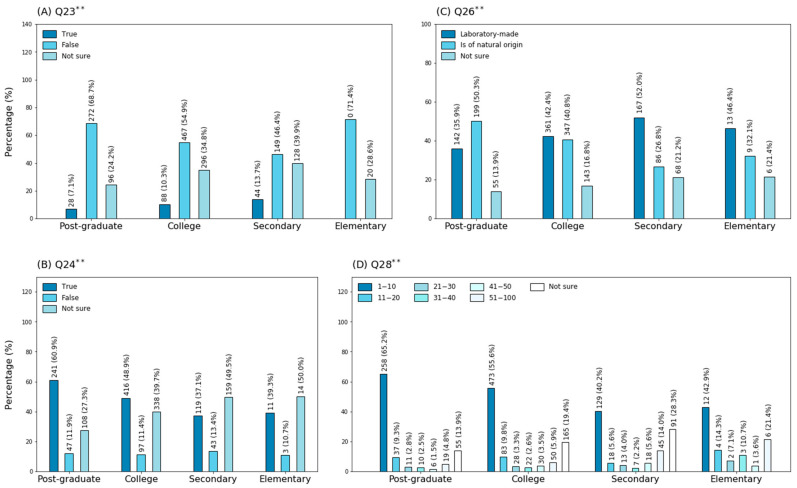
Comparison of response percentages among educational group: (**A**) Question 23; (**B**) Question 24; (**C**) Question 26; (**D**) Question 28. ** Chi-square values significant at *p* < 0.01.

On the other hand, around half of the population (56%) were not sure whether the virus is observable under a common microscope or not (Table 3). Such knowledge was more common in people with postgraduate instruction than in those with college (68.7%) and secondary education (49%); significant differences were found only by educational attainment (Figure 3A).

Likewise, only 49% of participants affirmed that the agent responsible for COVID-19 is an RNA virus (Table 3). This information was more popular among individuals with postgraduate studies (60%) than among those belonging to the other categories, as only 50% of participants with college, secondary and elementary education could answer adequately; significant differences were revealed only in relation to the educational level (Figure 3B). Interestingly, only 40.2% of the interviewees declared they considered the virus of natural origin, while 42.8% thought it was created in a laboratory and the remaining 17% stated they were not sure (Figure 2). The notion that the virus is man-made seemed most common among people without postgraduate education, as around 50% of the individuals belonging to such categories selected that option; significant differences were only detected by educational attainment (Figure 3C). In addition, participants were not necessarily familiarized with the number of COVID-19-related deaths (per 100 individuals) in Ecuador, as only half of the interviewees chose the correct option (Figure 2). Sixty-five percent of participants with postgraduate instruction stated that one to ten patients die of COVID-19 complications. The percentage of participants selecting the appropriate response decreased in relation to the educational level, with 55% of college-educated participants answering adequately, and with around 40% of those with secondary and elementary education electing the correct alternative; chi-square analysis revealed significant differences in relation to the level of education (Figure 3D).

## 4. Discussion

SARS-CoV-2 is the virus responsible for the COVID-19 pandemic that has had overwhelming repercussions worldwide since its detection in late 2019. National health authorities are involved in developing radical strategies in an attempt to prepare and manage the public, and thus to counteract the devastating effects of the virus. To develop appropriate strategies to deal with COVID-19, authorities must focus on crucial factors within of the population, such as knowledge, attitudes and prevention. These areas have already been studied and reported on by various investigations using similar surveys [24,25,26]. However, these studies have recommended the expansion of the questionnaire in order obtain a more precise understanding of the actual knowledge of COVID-19 among the public. Henceforth, the present survey includes additional questions with the aim of gaining a deeper understanding of the public perspectives regarding the symptoms, detection, transmission, prevention and, most notably, knowledge of the virus. Attitudes and practices were not the object of this investigation, as these areas have already been explored among Ecuadorians [27].

Understanding COVID-19 knowledge in individuals tested for the virus (using RT-PCR or immunological tests) appears crucial, as these people have been required to take the test, either for job-related issues or for developing symptoms associated with infection. Consequently, the surveyed population could be considered relevant since participants might be involved in the spreading of the disease. Furthermore, in this investigation, an innovative approach was explored in which laboratories played an active role in increasing the literacy of the general population. As mentioned before, participants were given the correct responses after answering each question, thus the detected misconceptions could have been clarified to some extent. After responding to the questionnaire, individuals were arguably bestowed with more reliable information that could be disseminated within their communities. In fact, several participants not only mentioned feeling satisfied with the correct answers being revealed after each question, but they also felt more knowledgeable about the subject after responding to the survey (Table S3). This approach is expected to increase the knowledge and awareness of COVID-19 among the public, which appears crucial as inadequate information and poor attitudes have been associated with inefficient diagnoses, modest control practices of infection, and ultimately, the dissemination of the diseases [29,30].

The average knowledge score of participants was 22.5 ± 3, with a correct rate of 80.4%. A similar rate was reported by a previous investigation carried out among Ecuadorians (82.3%) [27] and Malaysians (80.5%) [26], although the reported knowledge proved to be lower than that of Chinese residents (90%) [24,25]. The knowledge of participants was considered sufficient in studies where the overall correct rate was higher than 90% [24,25], whereas knowledge was considered moderate in those where the rate was around 80% [26,27]. Consequently, knowledge in the sampled population could not be considered elevated, which demonstrates that substantial work must be done if COVID-19 literacy is to improve in the area. Some common misunderstandings can be highlighted when comparing the present results with those of the aforementioned studies. For instance, people were not completely sure about symptoms such as stuffy or runny nose being less common in COVID-19 patients, with less than 60% responding correctly to the related query. Similarly, less than 50% responded correctly to the question related to airborne transmission. Recognizing these common patterns in the misunderstandings about the disease will be useful for mounting more informative public campaigns.

The statistical analysis revealed that scores differed significantly across educational levels. Postgraduate-instructed individuals obtained better scores than the rest, although they represented only 24% of the sampled population. This could be related to a better discernment of the information available about transmission and complications regarding the disease, as higher educational attainment has been associated with better knowledge of other illnesses [31,32]. On the other hand, the scores from those with secondary and elementary education were among the lowest. This may suggest that information about the virus cannot be easily discerned by people with partial education. Moreover, misinformation is constantly being disseminated, especially via social media, which certainly could be, in part, responsible for the apparent confusion regarding some key aspects of the virus and the disease. However, college-educated participants scored significantly lower than those with postgraduate instruction; this has also been reported by a recent study conducted among Ecuadorians [27]. Another investigation showed that undergraduate students in Quito were not necessarily aware of some basic genetic concepts [33]. Educational levels have been previously associated with lower scores regarding COVID-19 knowledge. Nevertheless, significant differences have also been found among other demographic groups not registered herein, including occupation [27], general income [26] or marital status [24]. It is important to highlight that due to differences regarding questionnaires and scoring systems, comparisons should always proceed with caution.

Undoubtedly, the public has been influenced by information released through official channels, as this subpopulation proved to be well-informed about the symptoms, detection, transmission and prevention of the disease, although participants were less confident regarding zoonotic transmission, which has been observed previously among Ecuadorian citizens [27]. Nevertheless, these outcomes suggest that some items should be addressed urgently. Firstly, our results show that participants consider the use of traditional and unproven treatments against COVID-19 effective. Since the beginning of the pandemic, several empirical treatments and homemade remedies have been deemed effective to battle infection, notwithstanding the lack of supporting information and the disregarding of potential side effects [34,35]. In addition, the severe consequences left by the first COVID-19 outbreak have deteriorated optimistic attitudes among the public, which has prompted a rise in the popularity of holistic medicine (pseudo medicine) and pseudoscience [36].

Secondly, participants were not necessarily well-informed about the biology of the virus, which was irrespective of their educational attainment. These results are in line with those from a previous report showing that undergraduate students, enrolled in careers unrelated to life sciences, were not familiar with key genetic concepts, such as genes carrying instructions for making proteins or the number of chromosomes passing down to the next generation [33]. In particular, the fact that interviewees were not necessarily aware of SARS-CoV-2 being an RNA virus, or that they considered the virus to be observable through a common optic microscope, might generate an inaccurate assessment of the laboratory efforts required for virus detection. Interestingly, the majority of participants stated that the virus was created in a laboratory, which evidenced that people give credit to the theory of a man-made virus, instead of acknowledging more pragmatic views considering processes of natural selection [37,38]. Despite the fact that participants identify higher severity and mortality rates in the elderly and people with comorbidities, they overestimate the rate of infected individuals/deaths caused by SARS-CoV-2. Half of the interviewees were not sure, or stated the rate to be over 10%, while official sources claim this to be around 6.6% [17]. This might be related to the abundant uncertified information circulating, mainly, on social media. Thus, it is expected that established information networks, such as traditional newspapers and television programs, help disseminate only reliable information via these platforms. The inadequate level of knowledge about the virus, along with the constant bombardment with misinformation, may impede the public in developing a proper interpretation of the disease, and so be able to interpret correctly, for instance, diagnostic results. In general, studies aimed at assessing COVID-19 knowledge have revealed that people are familiar with symptoms, contagion and prevention strategies. However, they were not fully confident with regards to zoonotic transmission. Moreover, these studies did not include queries dealing with the origin of the virus, its characteristics, as well as the efficacy of unproven treatment (Table 4). The current survey shows that efforts should focus on improving the literacy of scientific concepts regarding the virus, its transmission and its treatment, as interviewees certainly lacked sufficient criteria and seemed apparently exposed to constant misinformation. Consequently, we suggest that basic concepts about the biology of the virus must be considered for developing more accurate educational campaigns by public health authorities. 

This investigation presents some limitations, nonetheless. The survey was conducted in Quito and Ibarra, as the laboratories are located in such cities. Thus, the outcomes are not generalizable to other regions of the country, especially rural regions. As aforementioned, this investigation sought to gather information for developing an initial understanding of COVID-19 knowledge among citizens tested for the virus. This study did not aim at generating data that could be generalized to other groups. In addition, the instrument employed in this study was adapted from a questionnaire used in previous investigations to assess the COVID-19 knowledge, attitudes and practices of residents in China and Malaysia [24,26]. As attitudes and practices have been already assessed in Ecuadorians [27], the present survey focused on measuring knowledge of the pandemic in a particular subpopulation at risk of contracting and spreading the disease. Despite the usefulness of the analysis, especially due to the limited time and urgency of the situation, a more thoroughly evaluated questionnaire must be developed for achieving a more reliable assessment of COVID-19 literacy. Further investigations should focus on different subpopulations, such as healthcare workers, factory workers and students, in which transmission might be associated with overcrowding, lack of quarantine facilities and environmental contamination.

## 5. Conclusions

This study provided a comprehensive analysis of COVID-19 knowledge in individuals at risk of contracting and transmitting the disease in the cities of Quito and Ibarra in Ecuador. These findings suggest that participants were knowledgeable about the symptoms, detection, transmission and prevention of COVID-19. However, common misconceptions regarding the virus’ origins, its composition, and unproven treatments proved to be popular within the sampled population, as reflected by the responses to the associated queries. Further health educational programs must consider such topics in order to aid the population increase their understanding of the disease and, most importantly, avoid being persuaded by inaccurate information. For instance, educating people about the mechanisms by which the virus originated, via natural selection, will be crucial for preventing practices that facilitate the appearance of future zoonotic events. Similarly, instructing people about the RNA composition of the virus, along with the characteristics and functions of RNA, will be important for the public to get a better understanding of the development of RNA vaccines for treating infectious diseases. In fact, this seems particularly relevant in the context of COVID-19, as the vaccines developed by companies such as Pfizer/BioNTech and Moderna are based on RNA technology. Undoubtedly, understanding the RNA structure and function will help people comprehend how mRNA-based therapeutic approaches target critical processes of the virus life cycle. Additionally, proper tutoring about the dangers of unproven treatments might help face the menace of fake cures promoted, mainly, on social media. Instructing the population about the risks of misusing drugs and chemical products will be helpful for the public to initiate proper safety measures that would counteract the deleterious effects of COVID-19 misinformation on public health. Finally, the approach presented herein, of utilizing diagnostic laboratories as active educators within the community, will definitely raise the level of literacy among the population, and will ultimately improve prevention practices and reduce the spread of contagious diseases.

## Figures and Tables

**Table 1:** Demographic characteristics of participants and scores by demographic variable.

Variables	Number of Participants	%	Mean Score (Maximum 28)	t/F	*p*
**Age**				0.79	0.50
<18	10	0.6	21.5 ± 3		
18–29	362	22.7	22.5 ± 3		
30–49	868	54.4	22.4 ± 3		
>50	356	22.3	22.6 ± 3		
**Gender**				0.09	0.92
Female	854	53.5	22.5 ± 3		
Male	742	46.5	22.5 ± 3		
**Educational attainment**				50.1	<0.001
Postgraduate	396	24.8	23.6 ± 2 ^a^		
College	851	53.3	22.5 ± 2 ^b^		
Secondary	321	20.1	21.1 ± 3 ^c^		
Elementary	28	1.8	20.5 ± 5 ^c^		
**City of residence**				0.41	0.94
Quito	1261	79.0	22.5 ± 2		
Ibarra	115	7.2	22.2 ± 3		
Santo Domingo	56	3.5	22.4 ± 3		
Other cities *	164	10.3	22.2 ± 3		

Different superscript letters show significant differences (*p* < 0.01). * Other cities: cities with fewer than 50 counts.

**Table 2:** Outcomes of the multiple linear regression on factors associated with low scores concerning COVID-19 knowledge.

Variables	Coefficient	Standard Error	*t*	*p*
**Age**				
>50 vs. 18–29	0.365	0.224	1.627	0.104
>50 vs. 30–49	−0.022	0.184	−0.117	0.907
>50 vs. <18	1.004	0.996	1.008	0.313
**Gender**				
Male vs. female	−0.120	0.148	−0.816	0.414
**Educational attainment**				
Postgraduate vs. college	−1.205	0.182	−6.617	<0.001
Postgraduate vs. secondary	−2.631	0.222	−11.846	<0.001
Postgraduate vs. elementary	−3.427	0.606	−5.655	<0.001
**City of residence**				
Quito vs. Santo Domingo de Tsáchilas	0.067	0.397	0.168	0.866
Quito vs. Ibarra	−0.174	0.283	−0.614	0.539
Quito vs. Other cities *	−0.368	0.241	−1.526	0.127

* Other cities: cities with fewer than 50 counts.

**Table 3:** True/false questions used for assessing knowledge of COVID-19.

	Counts True	%	Counts False	%	Counts Not Sure	%
**Symptoms**						
Q1. Unlike the common cold, stuffy nose, runny nose, and sneezing are less common in persons infected with the COVID-19 virus. (T)	925.0	58.0	368.0	23.1	303.0	19.0
Q2. The main clinical symptoms of COVID-19 are fever, fatigue, dry cough, and body aches. (T)	1534.0	96.1	35.0	2.2	27.0	1.7
Q3. Not all persons with COVID-2019 will develop severe cases. Only those who are elderly and have chronic illnesses are more likely to be severe cases. (T)	1563.0	97.9	17.0	1.1	16.0	1.0
**Detection**						
Q4. PCR is the most accurate technique for identifying the virus in a patient. (T)	1474.0	92.4	63.0	3.9	59.0	3.7
Q5. The rapid test is the most accurate technique for identifying the virus in a patient. (F)	90.0	5.6	1428.0	89.5	78.0	4.9
**Treatment**						
Q6. Currently, there is no effective cure for COVID-19, but early symptomatic and supportive treatment can help most patients recover from the infection. (T)	1519.0	95.2	31.0	1.9	46.0	2.9
Q7. Chlorine dioxide is considered safe to treat COVID-19. (F)	134.0	8.4	1112.0	69.7	350.0	21.9
Q8. Some natural alternatives such as honey, garlic, ginger or eucalyptus can be used to treat the disease/fight the virus. (F)	662.0	41.5	720.0	45.1	214.0	13.4
**Transmission**						
Q9. Eating or touching wild animals would result in infection by the virus. (F)	143.0	9.0	1259.0	78.9	194.0	12.2
Q10. Persons with COVID-19 cannot infect others if they do not have a fever. (F)	38.0	2.4	1497.0	93.8	61.0	3.8
Q11. The virus spreads via respiratory droplets of infected individuals. (T)	1535.0	96.2	17.0	1.1	44.0	2.8
Q12. People can be infected and contagious despite having no clear symptoms of the disease. (T)	1560.0	97.7	16.0	1.0	20.0	1.3
Q13. The virus is airborne transmitted. (F)	618.0	38.7	739.0	46.3	239.0	15.0
Q14. Officially, dogs and cats are considered of low risk for transmission. (T)	1081.0	67.7	378.0	23.7	137.0	8.6
**Prevention**						
Q15. To prevent the infection by COVID-19, individuals should avoid going to crowded places and avoid taking public transportation. (T)	1555.0	97.4	25.0	1.6	16.0	1.0
Q16. Isolation and treatment of people infected with the virus are considered effective ways to reduce the spread of the virus. (T)	1566.0	98.1	14.0	0.9	16.0	1.0
Q17. It is necessary for children and young adults to take measures to prevent infection by the virus. (T)	1545.0	96.8	34.0	2.1	17.0	1.1
Q18. People who have contact with someone infected with COVID-19 should be immediately isolated in a proper place, the isolation period being 14 days. (T)	1487.0	93.2	80.0	5.0	29.0	1.8
Q19. The use of gloves provides total security against infection. (F)	55.0	3.4	1469.0	92.0	72.0	4.5
Q20. Ordinary residents can wear face masks to prevent infection by the virus. (T)	1570.0	98.4	8.0	0.5	18.0	1.1
Q21. It is correct to return to the workplace without being tested for coronavirus. (F)	150.0	9.4	1353.0	84.8	93.0	5.8
Q22. People who work in public service should always wear protective screens to prevent infection. (T)	1511.0	94.7	49.0	3.1	36.0	2.3
**Knowledge on SARS-CoV-2**						
Q23. Coronavirus is observable with a common microscope. (F)	160.0	10.0	908.0	56.9	528.0	33.1
Q24. The agent responsible for COVID-19 is an RNA virus. (T)	787.0	49.3	190.0	11.9	619.0	38.8

**Table 4:** Summary of studies assessing knowledge of the COVID-19 pandemic.

Study Desing	Target Group	Correct Answer Rates	Summary of Findings	References
Cross-sectional	Ecuadorians	13.8–98.6%	Participants were familiar with symptoms, contagion and prevention strategies. However, they were not so confident regarding zoonotic transmission. No questions regarding the virus, its characteristics and origins along with the efficacity of unproven treatments were formulated. Attitudes and practices were also assessed.	[27]
Cross-sectional	Colombians	61.2–98.9%	Participants had low to moderate levels of knowledge about COVID-19. No questions regarding the virus, its characteristics and origins, along with the efficacy of unproven treatments, were formulated. Attitudes and practices were also assessed.	[39]
Cross-sectional	Malaysians	35.7–99.1%	Interviewees were knowledgable about symptoms, contagion and prevention strategies. Nevertheless, they did not respond correctly to the query dealing with zoonotic transmission. No questions regarding the virus, its characteristics and origins, along with the efficacy of unproven treatments, were formulated. Attitudes and practices were also assessed.	[26]
Cross-sectional	Chinese Residents	70.2–98.6%	All participants proved to have strong knowlegde about symptoms, contagion and prevention strategies. No questions regarding the virus, its characteristics and origins, along with the efficacy of unproven treatments, were formulated. Attitudes and practices were also assessed.	[24]
Cross-sectional	General population in Kano, Nigeria	49.48–81.28%	The majority of the participants had poor knowledge, attitudes and practices. Half of the participants were aware of the zoonotic nature of the disease. No questions regarding the virus, its characteristics and origins, along with the efficacy of unproven treatments, were formulated. Beliefs were also assessed.	[40]
Cross-sectional	Bangladeshis	50.4–96.7%	Participants were knowledgable on symptoms, with deficiencies on contagion and prevention strategies. There was a knowledge gap on COVID-19 being treatable. No questions regarding the virus, its characteristics and origins, along with the efficacy of unproven treatments, were formulated. Perception was also assesed.	[41]
Cross-sectional	Ecuadorians tested for the virus in Quito and Ibarra	45.1–98.4%	Participants had strong knowledge about symptoms, detection, transmission and prevention strategies, although they were dubious with regards to zoonotic transmission. Scores were lower in comparison to questions regarding the virus, its characteristics and origins, along with the efficacy of unproven treatments. Attitudes and practices were not assessed.	This study

## Data Availability

The data presented in this study are available on request from the corresponding authors.

## Supplementary Materials

The following are available online at https://www.mdpi.com/1660-4601/18/4/1691/s1, Table S1: Checklist for reporting results of internet e-surveys (CHERRIES); Table S2: Original questionnaire in Spanish and its translation into the English language; Table S3: Raw data.
