# Supplementary material for: An On-Line Cross-Sectional Questionnaire to Assess Knowledge of COVID-19 Pandemic among Citizens Tested for the SARS-CoV-2 Virus in Quito and Ibarra, Ecuador
Source: Int J Environ Res Public Health. 2021 Feb 10;18(4):1691. doi: 10.3390/ijerph18041691 (PMC7916451; doi:10.3390/ijerph18041691)
Supplement: Supplementary file 1 [file ijerph-18-01691-s001.zip › Suppl/Table S1.docx]

# Appendix

[**Supplementary file A1].**

# Checklist for Reporting Results of Internet E-Surveys (CHERRIES).

| **Checklist for Reporting Results of Internet E-Surveys (CHERRIES).** | |
| --- | --- |
|  | **Design.** |
| **Describe survey design.** | The target population of this questionnaire were the Ecuadorians tested for SARS-CoV-2. Our sample was a convenience sample. |
| **Institutional Review Board approval and informed consent process.** | |
| IRB approval. | This study used secondary data sources and did not collect personal information. IRB approval was not necessary. |
| Informed consent. | There was an informed consent at the beginning of the questionnaire. Participants were informed of the purpose of the study that no personal information was going to be collected. They were also informed of the length of the questionnaire. |
| Data protection. | The questionnaire did not collect personal information, so additional mechanisms to protect unauthorised access to those provided by the platform (Google Forms) were not needed. |
| **Development and pre-testing.** | |
| Development and testing. | The questionnaire was developed by the designers authors of the research (declared in author contributions), following the CHERRIES checklist and ensuring usability and technical functionality. For internal validation, statistical analysis were performed. |
| **Recruitment process and description of the sample having access to the questionnaire.** | |
| Open survey versus closed survey. | The questionnaire was a closed survey. |
| Contact mode. | The initial contact with the potential participants was made at the laboratory, when the test was required. |
| Advertising the survey. | Advertising the survey. The survey was not announced or advertised in the web. |
| **Survey administration.** | |
| Web/E-mail. | The e-survey was sent via e-mail to all the patients that signed the consent at the lab. |

1

| Context. | The survey was developed in Google Forms platform and sent via e-mail to all the patients that was tested for Sars-cov 2 at the lab and signed the consent for the use of the data submitted. |
| --- | --- |
| Mandatory/voluntary. | Completing and submitting the survey was voluntary. |
| Incentives. | No incentives were offered. |
| Timeframe. | The survey was open for 6 weeks. |
| Randomization of items or questionnaires. | Randomization of items was performed. |
| Adaptive questioning. | No adaptive questioning was needed. |
| Number of Items. | The questionnaire included 32 items: 6 sections (demographic, symptoms, knowledge regarding PCR versus rapid tests, treatments, transmission and prevention) |
| Number of screens (pages). | The questionnaire had 4 pages, including initial information. |
| Completeness check. | All items of the questionnaire were stated as required. Therefore, to submit a questionnaire all items should be answered. By consensus, the authors concluded that all the items, except the demographic section, of the questionnaire should include a «I am not sure» option. |
| Review step. | Respondents were able to review and change their answers through a Back button. |
| **Response rates.** | |
| Unique site visitors. | We no provide view rates or participation rates. |
| View rate (Ratio unique site visitors/unique survey visitors). | Google Forms did not provide the number of unique visitors of the first page of the survey. |
| Participation rate (Ratio unique survey page visitors/agreed to participate). | Google Forms did not provide this data. |
| Completion rate (Ratio agreed to participate/finished survey). | Not applicable, to submit a questionnaire all items should be answered. |
| **Preventing multiple entries from the same individual.** | |
| Cookies used. | No cookies were used to assign a unique user identifier to each client computer. As personal information was not collected, we could not prevent duplicates entries. |
| IP check. | The IP address of the client computer was not collected nor used to identify potential duplicate entries from the same user. |
| Log file analysis. | Other techniques to analyse the log file for identification of multiple entries were not used. |

| Registration. | No registration was needed. |
| --- | --- |
| **Analysis.** | |
| Handling of incomplete questionnaires. | Only completed questionnaires could be submitted. |
| Questionnaires submitted with an atypical timestamp. | Google Forms only provided a timestamp. |
| Statistical correction. | Indicate whether any methods such as weighting of items or propensity scores have been used to adjust for the non-representative sample; if so, please describe the methods. |
